# Dimensions of Horizontal Gene Transfer in Eukaryotic Microbial Pathogens

**DOI:** 10.1371/journal.ppat.1005156

**Published:** 2015-10-29

**Authors:** Emile Gluck-Thaler, Jason C. Slot

**Affiliations:** Department of Plant Pathology, Ohio State University, Columbus, Ohio, United States of America; Geisel School of Medicine at Dartmouth, UNITED STATES

## Introduction

Comparative genomic studies of microorganisms have disrupted the paradigm of vertical inheritance with modification. First in bacteria, and more recently in microscopic and even multicellular eukaryotes, horizontal gene transfer (HGT) has been implicated in genomic and ecological evolution. HGT is the exchange of genetic material between organisms that occurs independently of meiotic and mitotic recombination between mating or hybridizing individuals. HGT occurs as viral and plasmid-mediated transfer, and transformation by environmental DNA via known or yet-unknown mechanisms [1]. The existence of environmental gene pools and pan-genomes is supported by decades of functional and phylogenetic studies in bacteria that have highlighted the exchange and proliferation of virulence factors and antibiotic resistance mechanisms [2–4]. Presently, accumulating reports of HGT in eukaryotes raise similar questions of how exposure to such gene pools has impacted the evolution of eukaryotic microbes, and whether or not human activities influence HGT dynamics. Here, we describe the evidence supporting HGT in eukaryotic microbial pathogens from divergent lineages that impact human, animal, and plant health (S1 Table). We consider three interacting dimensions affecting the prevalence of HGT (genetic network structure, selectable functions, and opportunity for contact) in order to better understand how HGT manifests in this important group of organisms.

## Does HGT Really Contribute to Eukaryotic Microbial Pathogen Genomes?

Reports of HGT among eukaryotic microbial pathogens have accumulated in recent years, largely driven by comparative genomic analyses showing an unexpected distribution and phylogenetic placement of gene sequences. However, these analyses require appropriate sampling and methodology, and should be interpreted with caution. Objections to the veracity and extent of HGT include the absence of a reproducible transfer mechanism in some lineages, and the plausibility of alternative explanations for the distributions and phylogenies of HGT candidate gene sequences [5]. The alternative interpretations of unexpected gene distributions and phylogenies hinge on different assumptions about the parsimony of a small number of gene transfer events versus a large number of the more widely accepted processes of gene duplication and loss [5]. Proponents of HGT hypotheses emphasize the importance of robust phylogenetic analyses coupled with multiple additional lines of evidence to support claims [6,7]. The most convincing cases have thus combined model-based phylogenetic approaches, which compare the topologies of gene trees to species phylogenies, with additional support from genome structure, sequence identity, codon usage, GC nucleotide content, and evidence of benefits to the recipient (S1 Table). Analyses relying on but a few of these methods, especially supporting methods in isolation, are rarely sufficient to strongly support HGT, and can result in false positive identification of HGT [7]. Unsampled genetic diversity at the population and species levels, which can impact reconstructions of gene distribution and inheritance, may also lead to false positive identification of HGT, underscoring the importance of robust taxon sampling [8]. For example, in a 2011 study, genes encoding the greatly expanded Crinkler protein family in the amphibian pathogen *Batrachochytrium dendrobatidis* had a distribution and phylogeny consistent with HGT from plant pathogenic oomycetes; however, the recent detection of Crinkler homologs in two additional fungal lineages opens the possibility that the current distribution is compatible with vertical inheritance and widespread gene loss [9–11]. Similar sampling biases can also lead to the underestimation of HGT in lineages of eukaryotic microbial pathogens, due to recent HGTs that are not fixed in populations and transferred genes that are prone to subsequent loss under changing selective pressures. This is illustrated by the rapid, differential degeneration of a horizontally acquired gene cluster among members of the necrotrophic fungal genus *Botrytis* [12]. More thorough sampling efforts currently underway may reduce erroneous inferences about HGT, help resolve the timing and direction of HGT events, and provide better estimates of their ecological contexts [13].

## Does Genetic Network Complexity Influence HGT?

**Fig 1:**
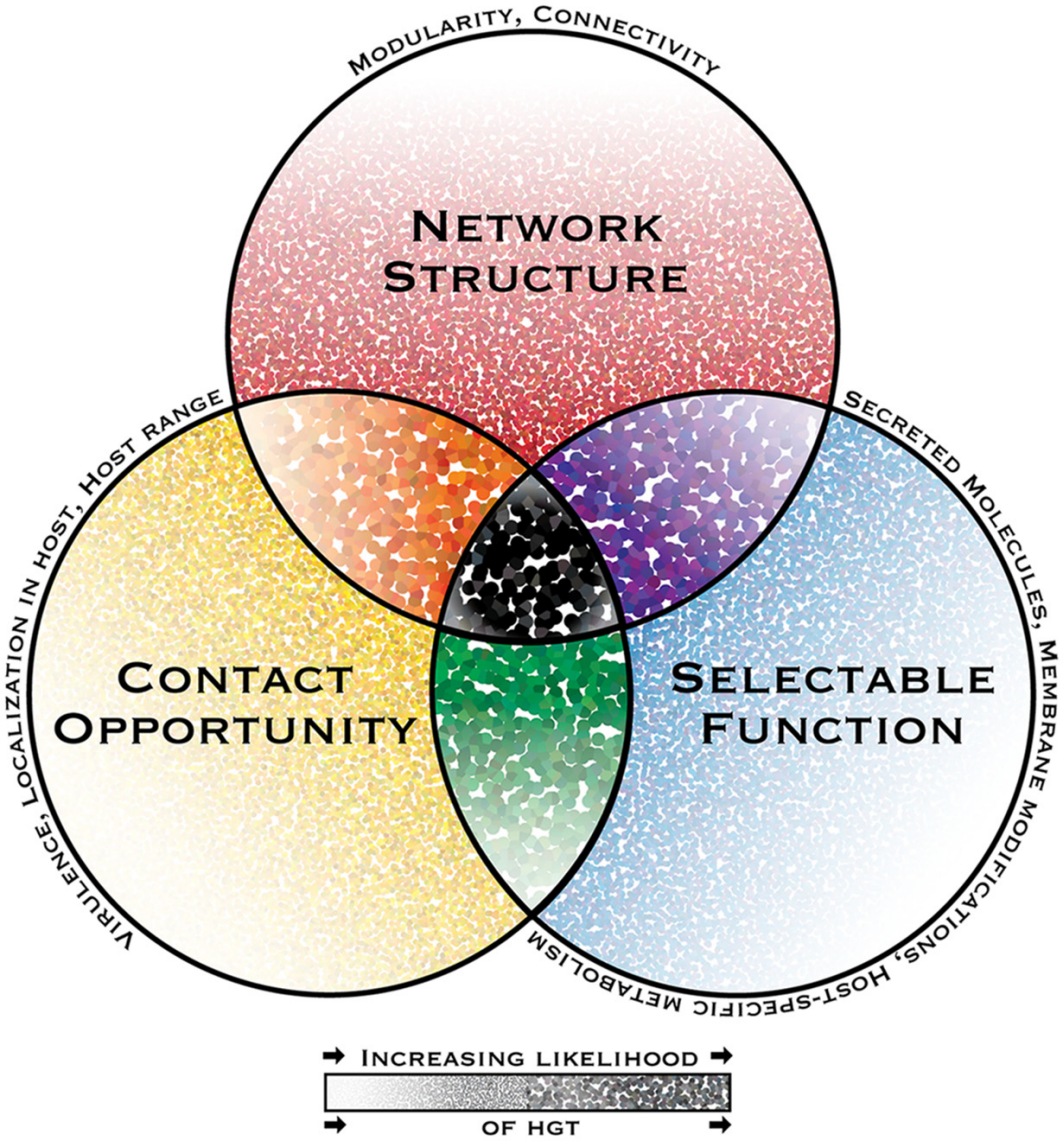
The interacting dimensions of horizontal gene transfer in eukaryotic microbial pathogens. The probability of horizontal gene transfer (HGT) and retention of a gene in recipient organisms is proposed to be under three main interacting influences or dimensions: (1) the genetic network structure, defined as the sum of functional connections between the gene and all other genes within a genome; (2) the selectability of the phenotype conferred by its function in a host environment; and (3) opportunity for contact, i.e., the rate and intimacy of meetings between donor DNA and recipient organisms throughout their life cycles. In this depiction of a general model, the probability of HGT increases with increasing dot intensity and size. Some pathogen-specific parameters influencing each dimension are listed above each circle’s perimeter. While HGT and the subsequent maintenance of genes in recipient genomes might be possible under the influence of only one or two dimensions, it is predicted to have the highest probability when all dimensions interact.

The complexity hypothesis posits that genes that are more modular in nature (i.e., residing at the periphery of gene connectivity networks) are more likely to be successfully transferred, because they are less disruptive to host networks and require establishment of fewer connections (Fig 1) [2,14]. This hypothesis is supported in bacteria, in which low network connectivity is found to enhance a gene’s “transferability,” which is largely independent of its specific biological function [15,16]. In eukaryotic microbial pathogens, genes encoding virulence factors may provide a fitness advantage to the recipient without extensive integration into genetic networks. Examples include genes encoding secreted effector proteins and specialized metabolic genes, especially those located in complete multigene clusters, which encode mechanisms for regulation, compartmentalization, secretion of products, and stoichiometric control of toxic intermediates [17–24]. Biased rates of gene transfer and loss of function found in systematic investigations of pathogen genomes also support the complexity hypothesis. Notably, in the human pathogen *Trichomonas vaginalis*, only 2% of 152 horizontally transferred genes were “informational” (more often involved in highly conserved, connected cellular processes like transcription), compared to 65% that encoded metabolic enzymes, which are “operational” genes associated with less conserved processes [25,26]. However, a survey of metabolic enzymes in select pathogens found that the degree of network connectivity of horizontally transferred genes was not different from the connectivity of vertically inherited genes [27]. The authors note that this could be because the horizontally transferred genes integrated into existing networks during the extensive time period since they were acquired [28].

A notable consequence of limited gene connectivity associated with horizontally transferred genes is the relaxation of network-imposed selection pressures, giving rise to sequence divergence, thus increasing the capacity for adaptive evolution. This effect has been reported in plant pathogenic *Pyrenophora* spp., in which ten horizontally transferred genes present significantly more diversifying sequence change compared to corresponding homologs in donor species [29].

## What Biological Functions Favor Successful HGT in Eukaryotic Microbial Pathogens?

Environmental selection in pathogen niches may favor the acquisition of certain gene functions. To date, there have been few formal investigations of general trends in functions of genes transferred to eukaryotic microbial pathogens (but see [6,30,31] for discussion of trends in fungi and oomycetes), and few functional confirmations of the roles horizontally transferred genes play in virulence [27,28]. Individual reports suggest three functional categories that are often horizontally transferred to divergent pathogen lineages: secreted molecules, membrane modifications, and metabolism specialized to host interactions and environments (Fig 1). Secreted molecule genes that have been transferred include those for degradative enzymes, such as the 22 different plant polysaccharide depolymerization enzymes that were transferred from phytopathogenic fungi to oomycetes [18]. Genes for production of toxic metabolites that disrupt normal cellular function are also reported transferred, including the fumonisin mycotoxin gene cluster between phytopathogenic *Fusarium* and *Aspergillus* fungi [22]. Membrane modifications identified in HGT reports may directly mediate cellular contact between hosts and pathogens, or mask pathogen membranes from host defense responses [32]. For example, a septin trans-membrane protein acquired by the microsporidian *Ordospora colligata* may facilitate the endocytosis-mediated infection of its *Daphnia* hosts [33]. Finally, the environment, i.e., the host, selects for the ability in the pathogen to metabolize and/or utilize host defenses and resources, or other sources of fitness in or on the host. For example, osmoregulatory genes acquired by phytopathogenic fungi may facilitate cellular osmotic balance in vascular fluids, and an anaerobic sulfur mobilization gene may increase survival of *Blastocystis* (a stramenopile suspected to be a pathogen) in anaerobic gut environments [19,20]. Some genes or gene clusters may be considered “repeat offenders,” having been transferred multiple times, possibly due to advantages conferred in specific pathogenic ecologies. For example, the complex distribution of epipolythiodioxopiperazine toxin gene clusters in ascomycete fungi suggests at least three independent transfers between divergent lineages [23].

The extent to which de novo gene gain promotes rapid pathogen emergence is largely unknown. One apparently contemporary transfer of a secreted toxin-encoding gene required for complete virulence on wheat, from *Stagonospora nodorum* to *Pyrenophora tritici-repentis*, has been reported [17]. The role of HGT in pathogen emergence is additionally supported by functional studies of transferred genes. For example, deletions of two horizontally acquired genes from the grass pathogen *Fusarium pseudograminearum*, and of a horizontally acquired osmoregulatory gene from vascular wilt fungi, resulted in reduced virulence [19,34]. Conversely, gain of virulence was documented in a fungal endophyte transformed with a membrane modification gene that the related entomopathogenic fungus, *Metarhizium robertsii*, may have ancestrally acquired from insects [32].

The fitness benefits conferred by horizontally acquired genes may range from none to highly beneficial, and may not directly relate to pathogenesis. Some pathogens exhibit complex life cycles that alternate between pathogenic and non-pathogenic states, and alternatively the gain of adaptive genes with no pathogenic function could facilitate attenuation of pathogenicity or transition to free-living status under selection from host density. For example, it was speculated that the transfer of a nitrate assimilation gene cluster to the mycoparasitic *Trichoderma* fungi may promote a transition to the nitrogen-limited wood-decay niche, and the transfer of a sugar utilization gene cluster to *Schizosaccharomyces* yeast could be part of an ecological transition from pathogen to fermenter [35,36].

## Do Eukaryotic Microbial Pathogens Become “Who They Meet?”

The frequency of physical contact between donors and recipients should be considered a driving force behind the likelihood of HGT events, and is a function of an organism’s ecology. Bacteria isolated from the same human body site, for example, exchange genes more frequently, and the genes they exchange are more frequently associated with niche-specific functions [3]. Three groups of ecologically adjacent organisms are often shown to be involved in horizontal gene exchange with eukaryotic microbial pathogens: co-infecting pathogens, non-pathogens symbiotically associated with the host, and the hosts themselves. Examples of transfers between potentially co-infecting pathogens include a host-specific toxin gene between two fungal wheat pathogens and a plant defense compound degradation cluster between fungal grass pathogens [17,21]. Non-pathogenic gut commensal bacteria are thought to have contributed diverse metabolic genes to *Trichomonas vaginalis* and *Blastocystis* genomes, including those involved in carbohydrate and amino acid metabolism [20,25,26]. Remarkably, there are cases of host-gene acquisition by insect- and plant-pathogenic fungi. These include the acquisition of a sterol binding protein by the entomopathogenic fungus *Metarhizium robertsii* that enables it to incorporate host-derived cholesterol into its cell membrane during infections, and the acquisition of a purine salvage pathway gene by obligate intracellular microsporidian pathogens, among others [32,33,37,38]. Considering the range of genetic exchange between ecological associates outside of predator–prey relationships, we suggest that the “you are what you eat” hypothesis proposed as a mechanism of HGT in phagotrophic eukaryotes may be rebranded “you are who you meet” for eukaryotic microbial pathogens [39]. We propose that the frequency of meetings between pathogens and specific classes of organisms may be influenced by virulence, localization in host, and host range (Fig 1). Less virulent pathogens may have sustained encounters with genomes of co-occurring pathogens, non-pathogenic symbionts, and the host because of their low impact on host mortality. In contrast, increasingly virulent pathogens may disproportionately encounter genomes from the greater environment as increased virulence correlates with increased survival time outside of hosts [40]. Obligate intracellular pathogens might be more frequently exposed to host genomes, while extracellular pathogens may often encounter genomes of other host-associated organisms. Similarly, generalists encounter a greater diversity of host genomes compared to specialists, and facultative pathogens may encounter more genes from the greater environment. Factors that contribute to a net increase in exposure to foreign DNA may favor acquisition of novel adaptive functions or drive an HGT ratchet by replacing pathogen genes with foreign genes (as proposed by Doolittle [39]). Furthermore, the gradually converging ecologies resulting from successive “meetings” may promote further transfers of ecology-specific genes such that decreasing ecological proximity results in acceleration of gene acquisition and vice versa. This could explain in part the repeated transfers of phytopathogenic genes from fungi to oomycetes, as well as the repeated acquisition of genes by the plant pathogenic *Fusarium* lineage from other plant pathogenic fungi, including the *Verticillium*, *Aspergillus*, and *Collectotrichum* genera [18,19,22,31,34]. It remains to be investigated whether specific pathogen lineages, virulence levels, host localizations, or specializations are more prone to horizontal gene exchange, but we note that lineage-specific biases in the rates of HGT were recently shown in a large comparative analysis of fungi [28].

## Do Human Activities Impact HGT in Eukaryotic Microbial Pathogens?

Human activities that accelerate environmental changes may impose selection pressures and precipitate dispersal events, which can influence the likelihood of HGT among eukaryotic microbial pathogens. Strong selection pressures exerted by decreased host diversity and intensive management practices in agro-ecosystems, including antimicrobials and other chemical control agents, may be expected to increase the prevalence of horizontally transferred genes, similar to the horizontal proliferation of antibiotic-resistant genes in bacteria due to modern overuse [4]. Homogeneous host environments increase the density of host-specific pathogens, non-pathogens, and the opportunities for them to interact, while at the same time relaxing density-dependent selection against virulent pathogens. Other human activities, such as global trade and travel, influence both the frequency and diversity of the close physical encounters required for horizontal gene flow, which can lead to emergence and evolution of pathogens in the near-term [17]. Furthermore, the migration of host ranges associated with climate and land-use changes provides new opportunities for encounters between pathogens established in previously isolated environments [41]. The extent of human impact on HGT in eukaryotic microbial pathogens is not known, but recent HGT discoveries in these organisms argue for careful consideration of pathogen emergence by HGT as a consequence of ecosystem management.

## Supporting Information

S1 Table Well-supported reports of HGT in eukaryotic microbial pathogens. This table details 21 references with at least one report of HGT among eukaryotic microbial pathogens. Recipient lineage, donor lineage, detection methods, putative contact opportunity, and information on gene functions are listed.
(PDF)
