# Supplementary material for: Dimensions of Horizontal Gene Transfer in Eukaryotic Microbial Pathogens
Source: PLoS Pathog. 2015 Oct 29;11(10):e1005156. doi: 10.1371/journal.ppat.1005156 (PMC4626037; doi:10.1371/journal.ppat.1005156)
Supplement: S1 Table — This table details 21 references with at least one report of HGT among eukaryotic microbial pathogens. Recipient lineage, donor lineage, detection methods, putative contact opportunity, and information on gene functions are listed. (PDF) [file ppat.1005156.s001.pdf]

Supplementary Table 1: Well supported reports of HGT in Eukaryotic Microbial Pathogens

Reference	Number of genes	Recipient lineage	Donor lineage	Detection methods	Putative contact opportunity	Putative selectable function within a pathogenic lifestyle	Expressed?	Contribution to virulence?
(1)	16	Apicomplexa	Bacteria	ML*	Unknown	Host-specific metabolism, others unknown	Unknown	Unknown
	24	Trypanosomatid	Bacteria	ML*	Unknown	Host-specific metabolism, others unknown	Unknown	Unknown
	17	Oomycota	Bacteria	ML*	Unknown	Host-specific metabolism, others unknown	Unknown	Unknown
(2)	1	*Blastocystis*	Bacteria	ML$^{\Psi}$, BI$^{\Psi}$	Non-pathogenic symbiont	Host-specific metabolism	Yes	Unknown
(3)	1	Fungi	Bacteria	ML$^{\Psi}$	Non-pathogenic symbiont	Host-specific metabolism	Yes	Unknown
(4)	6-gene cluster	Fungi	Fungi	ML$^{\Psi}$	Co-infecting pathogen	Secreted molecule	Yes	Unknown
(5)	3	Fungi	Bacteria	ML$^{\Psi}$, HS, GS, GC	Non-pathogenic symbiont	Host-specific metabolism	Yes	Yes
	2-gene cluster	Fungi	Fungi	ML* HS, GS	Unknown	Host-specific metabolism	Yes	Yes
(6)	7-gene cluster	Fungi	Fungi	ML$^{\Psi}$, TT, GS	Co-infecting pathogen	Host-specific metabolism	Unknown	Unknown
(7)	1	Fungi	Fungi	HS, PD	Co-infecting pathogen	Secreted molecule	Yes	Yes
(8)	5 clustered genes	Fungi	Fungi	ML$^{\Psi}$, GS	Co-infecting pathogen	Secreted molecule	Yes	Unknown
(9)	3	Fungi	Bacteria	BI*	Unknown	Host-specific metabolism	Unknown	Unknown
	2	Fungi	Metazoa/Fungi	BI*	Unknown	Host-specific metabolism	Unknown	Unknown
(10)	1	Fungi	Arthropoda	ML$^{\Psi}$, BI$^{\Psi}$, HS	Host	Host-specific metabolism	Unknown	Unknown
(11)	1	Fungi	Planta	ML$^{\Psi}$, TT, HS, SS, DC	Host	Secreted molecules	Yes	Unknown
(12)	15	Fungi	Bacteria	ML$^{\Psi}$, BI$^{\Psi}$	Non-pathogenic, other unknown	Host-specific metabolism, others unknown	Unknown	Unknown
	1	Fungi	Planta	ML$^{\Psi}$, GC, CUB	Host	Secreted molecule	Unknown	Unknown
(13)	6 clustered genes	Fungi	Fungi	ML*, TT, GS	Unknown	Secreted molecule	Unknown	Unknown
(14)	1	Fungi	Arthropoda	ML$^{\Psi}$, BI$^{\Psi}$, TT, GS	Host	Membrane modification	Yes	Yes
(15)	1	Fungi	Animalia	ML$^{\Psi}$	Host	Membrane modification	Unknown	Unknown
(16)	1	Fungi	Fungi	ML*	Unknown	Host-specific metabolism	Unknown	Unknown
	2	Fungi	Bacteria	ML$^{\Psi}$	Unknown	Secreted molecule	Unknown	Unknown
	6-gene cluster	Fungi	Fungi	ML*, GS, CUB	Non-pathogenic symbiont	Secreted molecule	Yes	Unknown
(17)	34	Oomycota	Fungi	ML*, TT	Unknown	Secreted molecule , host-specific metabolism, others unknown	Yes, others unknown	Unknown
(18)	96	Amoebozoa	Bacteria	BI*, HS, DM*	Unknown	Host-specific metabolism, others unknown	Unknown	Unknown
(19)	DNA segment containing 27 genes	Trichomonad	Bacteria	MP$^{\Psi}$, HS, PD, GS, GC, CUB	Non-pathogenic symbiont	Unknown	Unknown	Unknown
(20)	152	Trichomonad	Bacteria	BI*, DM*	Non-pathogenic symbiont, others unknown	Host-specific metabolism, others unknown	Unknown	Unknown
(21)	50	Trypanosomatid	Bacteria	BI*, DM*	Unknown	Host-specific metabolism, others unknown	Unknown	Unknown

* variable node support

$^{\Psi}$ greater than 2 nodes with strong bootstrap/posterior support

BI=Bayesian phylogenetic inference; ML=Maximum likelihood phylogenetic inference; TT=Statistical testing of alternative phylogenetic tree topologies; HS=Remarkably high sequence similarity; GS=Genome structure comparisons; GC=Analysis of GC content in DNA; PD=Analysis of >5 isolates for HGT presence; SS=Structural similarity of 3D protein; DC=Protein domain comparisons; CUB=Analyses of codon usage bias; MP=Maximum parsimony phylogenetic inference

References

1. Whitaker JW, McConkey GA, Westhead DR (2009) The transferome of metabolic genes explored: analysis of the horizontal transfer of enzyme encoding genes in unicellular eukaryotes. Genome Biology 10.
2. Tsaousis AD, Ollagnier de Choudens S, Gentekaki E, Long S, Gaston D, et al. (2012) Evolution of Fe/S cluster biogenesis in the anaerobic parasite Blastocystis. Proc Natl Acad Sci USA 109: 10426-10431.
3. Klosterman SJ, Subbarao KV, Kang S, Veronese P, Gold SE, et al. (2011) Comparative genomics yields insights into niche adaptation of plant vascular wilt pathogens. PLoS Pathog 7: e1002137.
4. Campbell MA, Staats M, van Kan JA, Rokas A, Slot JC (2013) Repeated loss of an anciently horizontally transferred gene cluster in *Botrytis*. Mycologia 105: 1126-1134.
5. Gardiner DM, McDonald MC, Covarelli L, Solomon PS, Rusu AG, et al. (2012) Comparative pathogenomics reveals horizontally acquired novel virulence genes in fungi infecting cereal hosts. PLoS Pathog 8: e1002952.
6. Greene GH, McGary KL, Rokas A, Slot JC (2014) Ecology drives the distribution of specialized tyrosine metabolism modules in fungi. Genome biology and evolution 6: 121-132.
7. Friesen TL, Stukenbrock EH, Liu Z, Meinhardt S, Ling H, et al. (2006) Emergence of a new disease as a result of interspecific virulence gene transfer. Nat Genet 38: 953-956.
8. Khaldi N, Wolfe KH (2011) Evolutionary origins of the fumonisin secondary metabolite gene cluster in *Fusarium verticillioides* and Aspergillus *niger*. International journal of evolutionary biology 2011.
9. Pombert JF, Selman M, Burki F, Bardell FT, Farinelli L, et al. (2012) Gain and loss of multiple functionally related, horizontally transferred genes in the reduced genomes of two microsporidian parasites. Proc Natl Acad Sci USA 109: 12638-12643.
10. Selman M, Pombert J-F, Solter L, Farinelli L, Weiss LM, et al. Acquisition of an animal gene by microsporidian intracellular parasites. Current Biology 21: R576-R577.
11. Armijos Jaramillo VD, Vargas WA, Sukno SA, Thon MR (2013) Horizontal transfer of a subtilisin gene from plants into an ancestor of the plant pathogenic fungal genus *Colletotrichum*. PLoS ONE 8: e59078.
12. Sun B-F, Xiao J-H, He S, Liu L, Murphy RW, et al. (2013) Multiple interkingdom horizontal gene transfers in pyrenophora and closely related species and their contributions to phytopathogenic lifestyles. PLoS ONE 8: e60029.
13. Patron NJ, Waller RF, Cozijnsen AJ, Straney DC, Gardiner DM, et al. (2007) Origin and distribution of epipolythiodioxopiperazine (ETP) gene clusters in filamentous ascomycetes. BMC Evol Biol 7: 174.
14. Zhao H, Xu C, Lu H-L, Chen X, St. Leger RJ, et al. (2014) Host-to-pathogen gene transfer facilitated infection of insects by a pathogenic fungus. PLoS Pathog 10: e1004009.
15. Pombert J-F, Haag KL, Beidas S, Ebert D, Keeling PJ (2015) The *Ordospora colligata* genome: evolution of extreme reduction in microsporidia and host-to-parasite horizontal gene transfer. mBio 6.
16. Dhillon B, Feau N, Aerts AL, Beauseigle S, Bernier L, et al. (2015) Horizontal gene transfer and gene dosage drives adaptation to wood colonization in a tree pathogen. Proceedings of the National Academy of Sciences.
17. Richards TA, Soanes DM, Jones MDM, Vasieva O, Leonard G, et al. (2011) Horizontal gene transfer facilitated the evolution of plant parasitic mechanisms in the oomycetes. Proc Natl Acad Sci USA 108: 15258-15263.
18. Loftus B, Anderson I, Davies R, Alsmark UC, Samuelson J, et al. (2005) The genome of the protist parasite *Entamoeba histolytica*. Nature 433: 865-868.
19. Strese A, Backlund A, Alsmark C (2014) A recently transferred cluster of bacterial genes in *Trichomonas vaginalis*-lateral gene transfer and the fate of acquired genes{Strese, 2014 #48}. BMC Evol Biol 14: 119.
20. Carlton JM, Hirt RP, Silva JC, Delcher AL, Schatz M, et al. (2007) Draft genome sequence of the sexually transmitted pathogen *Trichomonas vaginalis*. Science 315: 207-212.
21. Berriman M, Ghedin E, Hertz-Fowler C, Blandin G, Renauld H, et al. (2005) The genome of the African trypanosome *Trypanosoma brucei*. Science 309: 416-422.
